# The Role of Aminopeptidase ERAP1 in Human Pathology—A Review

**DOI:** 10.3390/cimb46030107

**Published:** 2024-02-20

**Authors:** Laura Țiburcă, Dana Carmen Zaha, Maria Claudia Jurca, Emilia Severin, Aurora Jurca, Alexandru Daniel Jurca

**Affiliations:** 1 Faculty of Medicine and Pharmacy, University of Oradea, Universității Street 1, 410087 Oradea, Romania; laura_tiburca@yahoo.com (L.Ț.); danaczaha@gmail.com (D.C.Z.); aurora.jurca@yahoo.com (A.J.); alexjurca@yahoo.co.uk (A.D.J.); 2 Regional Center of Medical Genetics Bihor, County Emergency Clinical Hospital, Bihor, 65–67, Gheorghe Doja Street, 410169 Oradea, Romania; 3 Department of Genetics, Carol Davila University of Medicine and Pharmacy, Dionisie Lupu 37 Street, 020021 Bucharest, Romania

**Keywords:** aminopeptidases, *ERAP1*, *ERAP2*, *LNEP*, major histocompatibility complex

## Abstract

Aminopeptidases are a group of enzymatic proteins crucial for protein digestion, catalyzing the cleavage of amino acids at the N-terminus of peptides. Among them are *ERAP1* (coding for endoplasmic reticulum aminopeptidase 1), *ERAP2* (coding for endoplasmic reticulum aminopeptidase 2), and *LNPEP* (coding for leucyl and cystinyl aminopeptidase). These genes encoding these enzymes are contiguous and located on the same chromosome (5q21); they share structural homology and functions and are associated with immune-mediated diseases. These aminopeptidases play a key role in immune pathology by cleaving peptides to optimal sizes for binding to the major histocompatibility complex (MHC) and contribute to cellular homeostasis. By their ability to remove the extracellular region of interleukin 2 and 6 receptors (IL2, IL6) and the tumor necrosis factor receptor (TNF), *ERAP1* and *ERAP2* are involved in regulating the innate immune response and, finally, in blood pressure control and angiogenesis. The combination of specific genetic variations in these genes has been linked to various conditions, including autoimmune and autoinflammatory diseases and cancer, as well as hematological and dermatological disorders. This literature review aims to primarily explore the impact of *ERAP1* polymorphisms on its enzymatic activity and function. Through a systematic examination of the available literature, this review seeks to provide valuable insights into the role of *ERAP1* in the pathogenesis of various diseases and its potential implications for targeted therapeutic interventions. Through an exploration of the complex interplay between ERAP1 and various disease states, this review contributes to the synthesis of current biomedical research findings and their implications for personalized medicine.

## 1. Introduction

Aminopeptidases are crucial enzymes distributed throughout the body, present both in cell membranes and subcellular organelles within the cytoplasm, playing diverse and intricate roles in cellular functions. Their primary function revolves around catalyzing the removal of amino acids from the amino terminus of protein chains. This process is pivotal in adapting longer peptide precursors to the appropriate size for presentation to molecules within the major histocompatibility complex (MHC) classes [1]. The MHC encodes molecules like Human Leukocyte Antigen (HLA) classes I and II, consisting of over 145,884 alleles. HLA class I molecules are involved in presenting antigens to CD8+ T cells, while HLA class II molecules perform the same function for CD4+ T cells. Notably, three genes, namely *ERAP1*, *ERAP2*, and *LNPEP*, hold significant roles in human pathology, especially concerning autoimmune diseases. These genes are situated on the long arm of chromosome 5 (5q15) and span approximately 200 kb, exhibiting quasi-identical roles [2].

The purpose of this article is to offer an in-depth review, drawing from the latest literature, to shed light on the involvement of the *ERAP1* gene in seemingly unrelated pathologies. Through an extensive exploration, this review aims to familiarize the readers with a gene that is often overlooked but holds multifaceted roles in human pathology, particularly in autoimmune and autoinflammatory diseases.

## 2. Genetics

**Figure 1:**
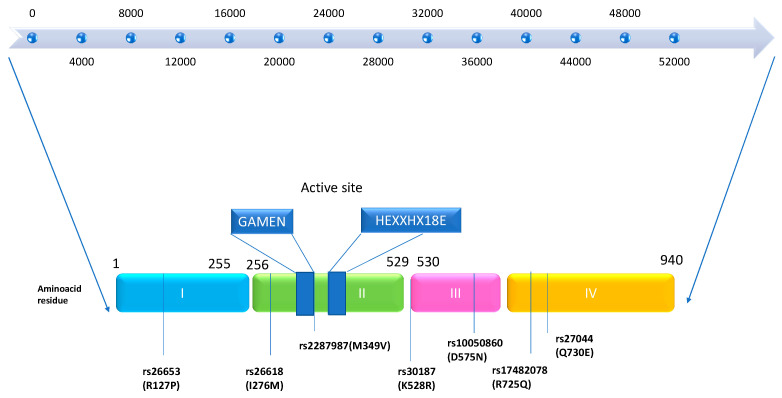
Domain structure of *ERAP1* gene and protein with autoimmune-associated SNPs: the ERAP1 gene spans 48kb in length. The ERAP1 protein consists of four domains: Domain I shown in light blue, Domain II in green, Domain III in pink, and Domain IV in orange. The active site of Domain II, containing GAMEN and HEXXHX motifs, is highlighted in dark blue. Single-nucleotide polymorphisms (SNPs) associated with autoimmune pathology are indicated with thin blue underlines. This figure was adapted from figure in [7].

The genes for endoplasmic reticulum aminopeptidase 1 (*ERAP1*) and *ERAP2* are located on chromosome 5q15. The discovery of the *ERAP1* gene was initially documented by the Wellcome Trust Case Control Consortium in 2007. It spans a length of 48 kb and comprises 19 exons and 18 introns, encoding the ERAP1 protein. Nguyen et al., in 2011, demonstrated through X-ray crystallography studies that the encoded protein, ERAP1, has a crystal structure. This protein consists of four domains: Domain I is a sandwich domain; Domain II represents the catalytic domain, serving as the active region of the protein and containing the motifs GAMEN and HEXXH-(X18)-E. Domain III is a small β-sandwich domain situated between Domains II and IV. Domain IV represents the regulatory domain, which is the most variable and acts as a protective shield for Domain II in a closed state. Removal of Domain IV from the protease zone of Domain II allows the substrate to gain access to the active site of the protein [3,4,5]. Conformational changes, including the reorientation of amino acid side chains in the active site and domain rearrangements, govern the transition between the open and closed states of ERAP1, thus influencing its activity (see Figure 1) [6,7].

Several important amino acid residues are located near the catalytic site in Domain II. Among these are residues 346 and 349 (rs2287987). Additionally, the peptide-binding site in Domain IV contains residues 725 (rs17482078) and 730 (rs27044), while the interface between Domain II and III includes residue 528 (rs30187). Furthermore, in Domain III, residue 575 (rs17482078) plays a role in enzymatic activity through conformational changes, affecting the transition between the closed and open forms of ERAP1 [8,9].

## 3. Function of ERAP1 and ERAP2

ERAP1 and ERAP2 are aminopeptidases that exhibit distinct but complementary properties and play a significant role in shaping the immunopeptidome of major histocompatibility complex I (MHC I). While ERAP1 cleaves the N-terminal hydrophobic residues, ERAP2 has an affinity for positively charged amino acids. This process is mediated by interferon-gamma (IFN-gamma). The mechanism is essentially as follows: peptides from the cytoplasm are translocated to the endoplasmic reticulum (ER) by the Transporter associated with Antigen Processing (TAP), a member of the ABC transporter family. Here, ERAPs trim their N-terminal ends to optimal lengths (8–10 amino acids). The resulting peptide–HLA class I complexes are transported to the cell surface and presented to CD8 T cells and NK cells, facilitating immune responses [10,11].

Abnormal trimming processes can lead to the formation of unstable HLA B27–peptide complexes, causing misfolding, accumulation of HLA-B27 heavy chains, and triggering the unfolded protein response (UPR), the IL-1 mediated inflammatory response, and tumor necrosis factor-alpha (TNF-α) production. This explains the persistence of inflammatory processes [12].

ERAP1′s involvement extends beyond antigen presentation. Reeves et al. demonstrated that ERAP1 also participates in shedding cytokine receptors on cell surfaces (e.g., IL-1R2, TNFR1, and IL-6Rα). This shedding reduces the ability of receptors to transmit signals, promoting inflammatory processes. Cytokines are known to regulate biological processes, including cytotoxicity, inflammatory and antiviral responses, and gene transcription [7].

Mutations in the ERAP1 gene can alter peptide trimming or antigen presentation, leading to a predisposition to autoimmune and autoinflammatory diseases and impairing immune responses by NK cells [2,13].

ERAP1 also plays a role in regulating innate immunity. It is primarily localized in the ER but can also be found as a soluble protein in the cytoplasm and as an integral type II membrane protein on the cell membrane. Depending on environmental changes, cellular stressors, and cell type, ERAP1 and ERAP2 may change their subcellular locations. For instance, inflammatory signals can influence their localization [14,15].

Proinflammatory cytokines IL-1β, IL-6, and TNF-α become more active, and NK and T cells experience increased activation when ERAP1′s peptide-trimming function is reduced, illustrating ERAP1’s role in modulating innate and adaptive immunity and causing inflammation.

**Figure 2:**
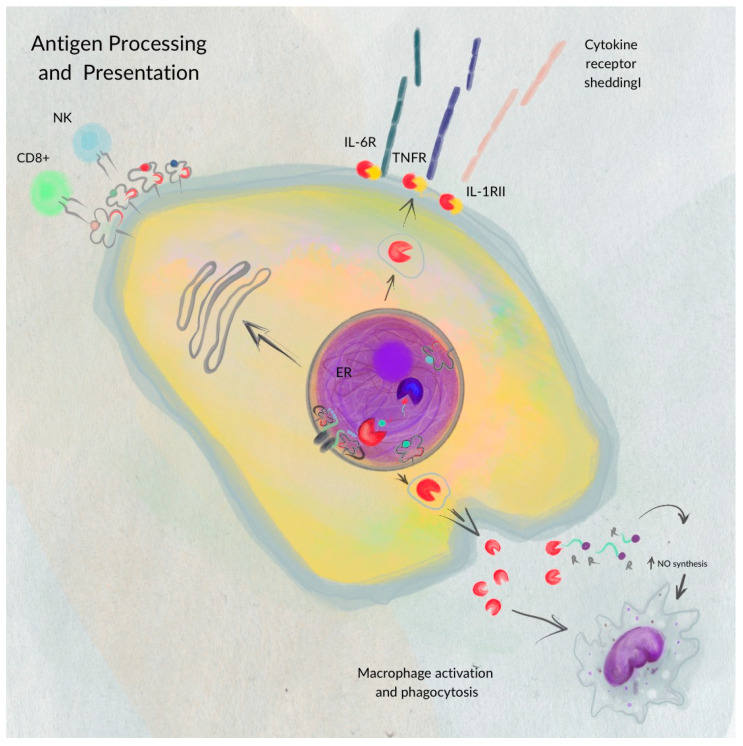
The polymorphic role of ERAP1—ERAP1 plays a polymorphic role in antigen processing for presentation on MHC I molecules at the cell membrane surface. Beyond its involvement in antigen presentation, ERAP1 also aids in the expression of cytokine receptors such as IL-6R, TNFR, and IL-1RII on the cell membrane surface, and it boosts macrophage activation and phagocytosis. This figure was adapted from figure in [7].

These findings demonstrate the polymorphic role of ERAP1 in both innate and adaptive immunostimulatory pathways (Figure 2), promoting inflammation through alternative pathogenic mechanisms, in addition to its well-characterized antigen processing function.

ERAP2 and ERAP1 have similar functions but may have slightly different preferences or efficiencies in cutting substrates at the N-terminal end and in processing peptides of various lengths. Specifically, ERAP2 tends to cleave positively charged residues, with its activity being highest on octameric substrates and decreasing on longer peptides such as 63, 72, 73, and 74. Thus, these two aminopeptidases would work together, ensuring effective generation and/or destruction of MHC class I epitopes for the proper functioning and regulation of adaptive immunity.

LNPEP (leucyl-cystinyl aminopeptidase) is responsible for processing endogenous peptides, such as arginine–vasopressin and oxytocin. Through splicing mechanisms, multiple transcripts are generated, leading to the production of various enzyme variants (isoforms). LNPEP plays diverse roles in organisms, including catalyzing the conversion of angiotensinogen into angiotensin IV (AngIV). It is also known as the AT4 receptor, expressed in various tissues (brain, heart, kidney, adrenal glands, and blood vessels), where it regulates blood flow and contributes significantly to neuroprotection. Moreover, it positively impacts several physiological and behavioral functions, influencing blood flow regulation, neuroprotection, synaptogenesis, long-term potentiation, memory consolidation, and recovery [3].

ERAP1 and LNPEP (IRAP) play crucial regulatory roles in blood pressure by inactivating angiotensin II (Ang II) and generating bradykinin, which are essential components of the renin–angiotensin system (RAS). The RAS has a vasoconstrictor function and is involved in regulating blood pressure, as well as maintaining the balance of sodium and potassium levels in the body (regulating sodium intake and potassium excretion). Moreover, angiotensin II (Ang II) is well known for its proinflammatory and profibrotic properties, highlighting its significant impact on various physiological processes [16,17].

The protein sequences of these aminopeptidases share a high degree of similarity, with LNPEP and ERAP1 showing 41–49% similarity and ERAP1 and ERAP2 having 49–50% similarity [3].

In summary, like ERAP1, LNPEP also contributes to antigen processing and presentation through MHC class I, regardless of TAP (Transporter associated with Antigen Processing) [3].

## 4. Role of ERAP1 in Human Pathogenesis

ERAP1 plays a significant role in the pathogenesis of various human diseases, including autoimmune diseases, cancer, atopic dermatitis, viral infections, and hematological disorders.

### 4.1. Autoimmune and Autoinflammatory Diseases

Autoimmune diseases constitute a large group encompassing over 80 conditions that share a common pathogenetic mechanism—an immune-mediated attack on the body itself. Although the individual incidence of each disease is relatively low, their combined prevalence is high, leading to conditions characterized by tissue damage resulting from an aberrant immune response [18]. In a comprehensive review published in 2022, Liu Shuang et al. explored the relationship between ERAP1 polymorphisms and autoimmune diseases, offering insights into how these variations may impact the development of new medications targeting both wild-type and mutant proteins with different polymorphisms [19]. These diseases are influenced by both genetic and environmental factors, following a multifactorial polygenic model of inheritance. They involve an inappropriate immune response in which the immune system erroneously attacks the body instead of providing protection. This category includes several conditions with multi-organ involvement, such as systemic lupus erythematosus, rheumatoid arthritis, Type 1 diabetes mellitus, multiple sclerosis (MS), and Graves’ disease. Autoimmune diseases are characterized by the presence of autoantibodies and auto-reactive T cells, reflecting an adaptive immune response [20].

On the other hand, autoinflammatory diseases manifest as episodes of apparent inflammation without an identifiable provoking cause, presenting symptoms like fever, skin rash, and arthritis. These conditions arise from defects in the inflammatory response regulated by the innate immune system, as observed in diseases like Crohn’s disease. Additionally, certain diseases display characteristics of both immune and autoinflammatory responses, including Behçet’s disease (BD), inflammatory bowel disease (IBD), ankylosing spondylitis (AS), and psoriasis (Ps) [20].

The immune system’s fundamental role is to recognize pathogens by identifying specific protein fragments, called antigenic peptides, on the cell surface. The ERAP1 and ERAP2 genes are known to play a pivotal role in the development of these immune-mediated diseases [3,21]. Genome-wide association studies (GWASs) have revealed a broad spectrum of loci associated with increased susceptibility to various autoimmune diseases. These studies have identified common gene combinations involved in inflammatory pathways, cytokines, cytokine receptors, and proteins related to T-cell activation [22,23].

Epistatic interactions between ERAP1 and specific HLA alleles have been reported in certain autoimmune conditions. For instance, in ankylosing spondylitis, HLA-B27; in psoriasis, HLA-C06; and in Behcet’s disease, HLA-B51. This highlights the significant role of antigen presentation in these conditions, collectively termed “MHC-I-opathy” [24,25].

**Figure 3:**
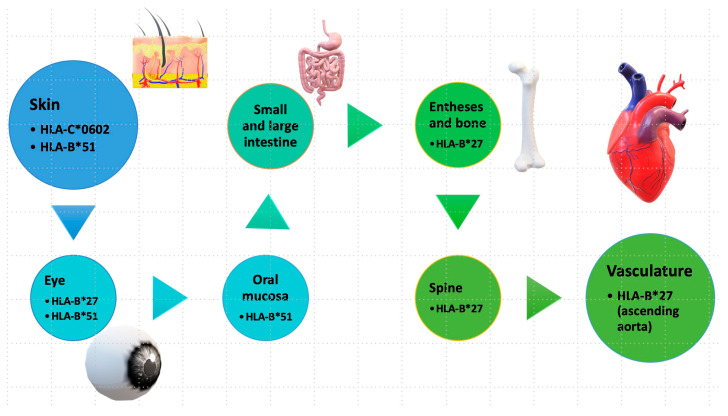
Tissue specificity in MHC-I-opathy. Antigenic structures at the level of different external or internal sites involved in MHC-I-opathies. * is used in Human Leukocyte Antigen (HLA) nomenclature to denote genetic polymorphisms or allelic variants within the respective HLA gene loci. https://hla.alleles.org/nomenclature/index.html.

The term “MHC-I-opathy” was introduced by McGonagle et al. in 2015 based on observations of immunopathological connections between Behcet’s disease and other spondyloarthropathies. The authors presented several arguments to support this concept, including shared immunopathogenic mechanisms, the involvement of tissue-specific factors triggering inflammatory responses, and the importance of unconventional lymphocytes, including innate lymphoid cells (ILCs) [26]. All these diseases have associations with the HLA system (Figure 3) and share common clinical features, such as localization in areas exposed to the external environment (oral mucosa, intestinal skin) and regions subject to physical stress, such as fibrous or fibrocartilaginous entheses (ocular, vascular walls). The phenotype may vary depending on how the organ responds to immune reactions, ranging from permanent blindness and spinal fusion to reversible skin changes between attacks [27].

#### 4.1.1. Ankylosing Spondylitis (AS)

Ankylosing spondylitis is a polygenic multifactorial disease that belongs to the larger group of spondyloarthropathies, which includes several conditions such as psoriatic arthritis, reactive arthritis, and arthritis associated with inflammatory bowel disease (IBD) [7]. Primarily affecting the spine, AS starts in the lumbar area but can progress to involve all segments of the spine, particularly affecting the sacroiliac joints early on. The main clinical symptom is joint inflammation, especially of the larger joints, accompanied by pain. The incidence of AS is 5 per 1000 European individuals, with a heritability rate of over 90%. The male-to-female sex ratio is 2:1, with a higher prevalence in males. AS shows a strong association with the HLA class I system [28].

The ERAP1 gene polymorphism has been extensively studied in numerous independent studies and ethnic populations due to its association with AS. ERAP1 is considered the second-strongest gene associated with AS, posing a risk factor for developing the disease.

The genetic link between the HLA-B27 system and AS is well documented, particularly with the subtypes HLA-B*27:02 and HLA-B*27:05. Gene polymorphisms in HLA-B27 influence the risk of AS, with susceptibility estimated at 26% [29,30]. Over 90% of AS patients are HLA-B27 positive, but only 1–5% of carriers develop the disease [31,32,33]. HLA-B27 contributes to the disease through three mechanisms: binding to arthritogenic peptides, abnormal presentation of heavy chains leading to the unfolded protein response (UPR), and formation of homodimers of B27 heavy chains not loaded with peptides [34,35].

There is a close correlation between the age of disease onset and HLA-B27, as demonstrated by the German spondylarthritis inception cohort (GESPIC). AS patients who are HLA-B27 positive are on average 8.5 years younger at disease onset compared to HLA-B27-negative patients [36,37].

**Figure 4:**
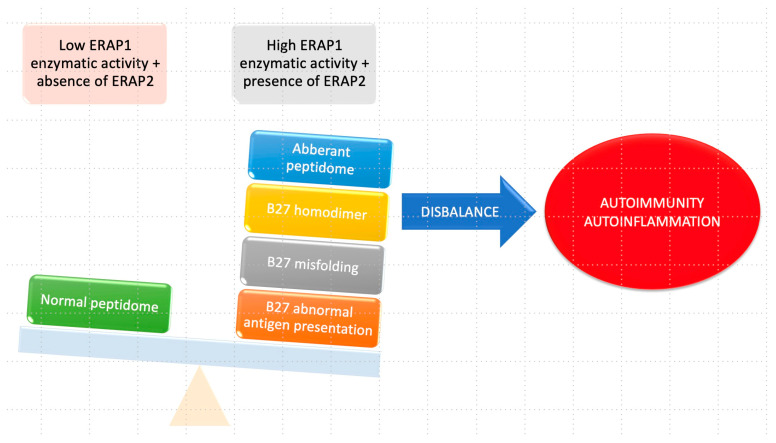
Relationship between HLA-B27 and ERAP1 in AS.

The complex relationship between HLA-B27 and ERAP1 in the context of ankylosing spondylitis (AS) is detailed in Figure 4. When the enzymatic activity of ERAP1 is increased, leading to improper cleavage of peptides, it results in the formation of an aberrant HLA–peptidome complex. This abnormal presentation of the antigen, combined with irregular folding, triggers the activation of autophagy. Furthermore, the formation of B27 homodimers occurs because of the abnormal HLA–peptidome complex. These B27 homodimers can activate killer immunoglobulin receptors (KIRs) and leukocyte immunoglobulin-like receptors (LILRs) in immune cells, contributing to the pathogenesis of AS.

In contrast, when HLA-B27 is paired with ERAP1 haplotypes characterized by low enzyme activity and in the absence of ERAP2, a normal peptidome will be present. This normal configuration ensures appropriate peptide processing, and as a result, the formation of B27 homodimers and subsequent activation of immunoglobulin receptors are minimized. The intricate interplay between HLA-B27 and ERAP1 plays a critical role in the development of ankylosing spondylitis, and their distinct enzymatic activities significantly influence the peptide presentation process and the subsequent immune responses involving immunoglobulin receptors [28].

In humans, the ERAP1 gene exhibits 10 known haplotypes (Hap1–Hap10), with a combined frequency of over 99% [8,38,39]. Currently, five SNP polymorphisms of the *ERAP1* gene have been identified: rs27044, rs30187, rs2287987, rs10050860, and rs17482078, which result in the amino acid substitutions M349V, K528R, D575N, R725Q, and Q730E, respectively (Table 1) [32,39]. Loss-of-function polymorphisms in ERAP1 influence the expression of free HLA-B27 heavy chains and disrupt the folding and presentation of peptides [2,40].

The SNP rs30187 (R528K) is the most involved variant, with the K528 minor allele variant (Hap1-3) being much more active, exhibiting a higher peptide-cutting rate compared to the R528 major allele variant. The major allele is associated with a protective role, while the minor allele is linked to an increased risk of disease. Notably, the association between rs30187 (K528R) and the minor allele rs10050860 (R725Q) has a protective role, reducing the risk of developing the disease by four times in individuals who are homozygous for these variants and HLA-B27 positive [27]. Additionally, SNP rs30187 is frequently associated with hypertension in patients with AS, a symptom often reported in individuals with this disease [6,41]. Haplotype III, which involves the presence of rs17482078/rs10050860/rs30187, increases susceptibility to the disease due to the improper cutting of HLA class I peptides [9].

In the context of family genetics, certain combinations of ERAP1/ERAP2 haplotypes can be identified, specifically the alleles rs27044-G and rs30187-T at the ERAP1 level, and the allele rs2549782-T at the ERAP2 level [6,42].

Studies in different populations have demonstrated an association between rs27044 and ankylosing spondylitis [43,44,45]. One issue that may arise in genotyping is the correct assignment of the SNP rs27044 allele. This polymorphism involves an allele change between G and C, which can create challenges in differentiating between sense and antisense strands.

In cases where the antisense chain is referred to, the major allele rs27044 is reported as the C sense allele [46]. Conversely, in another study, it was determined that the G allele is considered the major allele when the sense chain is taken as a reference [47,48].

Saad et al., in a recent study, outlined the significance of ERAP1 polymorphisms in axial spondyloarthritis (axSpA), indicating that their impact on axSpA may differ across diverse ethnic populations. Despite the established epistatic relationship between these polymorphisms and HLA-B27 in several studies, additional research is warranted across various ethnic backgrounds [49,50].

#### 4.1.2. Behçet’s Disease (BD)

Behçet’s disease (BD) is a multisystemic, self-inflammatory vasculitis of unknown origin. Its main symptoms include recurrent mouth and genital ulcers, as well as manifestations of eye, joint, blood vessel, and central nervous system involvement. The disease was named after Turkish doctor Hulusi Behçet, who first described it in 1937. The global prevalence of BD is approximately 10.3 cases per 100,000 individuals, with higher prevalence rates observed in countries in the Far East (Japan, China), the Middle East (Iran), and the Mediterranean basin (Turkey, Morocco, Tunisia), where rates reach 100–300 cases per 100,000 people in Turkey, 1 per 10,000 in Japan, and 0.3 per 100,000 in Northern Europe [51].

BD is often associated with the presence of HLA-B51, as well as with IL-10 and IL-23. Haplotype 10, which is linked to decreased enzymatic activity of ERAP1, is commonly found to be associated with HLA-B51 and is considered a major risk factor for BD, particularly in relation to the occurrence of epistaxis. Notably, among the ERAP1 gene polymorphisms, rs10050860 (D575N) and rs17482078 (R725Q) are most associated with BD patients, as well as with uveitis [52,53]. Interestingly, while these two polymorphisms have a protective role in ankylosing spondylitis (AS), they seem to increase susceptibility to BD [4,27,54].

Furthermore, the ERAP1-001 allotype, characterized by low shear activity, was shown by Takeuchi et al. in 2016 to increase the risk of BD in homozygous individuals. Specifically, HLA-B51 homozygous individuals carrying this allotype face an 11 times higher risk of developing BD [55,56].

#### 4.1.3. Birdshot Chorioretinopathy (BSCR)

Birdshot chorioretinopathy (BSCR) is an uncommon, chronic, progressive, ocular autoimmune disease that affects both eyes and primarily targets the retina and choroid. Due to its rarity, determining the exact incidence and prevalence of BSCR poses challenges. Patients with BSCR may experience irritation and keratic precipitation, although the anterior pole typically remains unaffected. In the posterior pole, multifocal areas of depigmentation may appear, accompanied by optical disc edema, narrowed retinal vessels, and cystoid macular edema.

The disease is characterized by multifactorial polygenic inheritance and has a strong correlation with the HLA allele A29:02, which belongs to the HLA class I group. In the general population, this allele is present in approximately 7%, but in individuals with BSCR, the prevalence is much higher, exceeding 95% [57]. Alvarez-Navarro et al. showed that changes in the cleavage mechanism mediated by ERAP1 can result in the formation of peptides longer than nine amino acids, with larger side chains and a higher affinity for HLA A29:02 ligands [58,59].

Additionally, a common polymorphism of the LNPEP gene (rs77050930) has been observed. Kuiper et al. conducted a genome-wide association study that involved 96 Dutch and 27 Spanish BSCR cases and 398 unaffected Dutch and 380 Spanish controls of European ancestry, analyzing a total of 9,932,851 SNPs in 117 cases and 693 controls. A common polymorphism of the LNPEP gene (rs77050930) was observed. Moreover, homozygotes for the risk allele rs10044354 C had reduced or no expression of the ERAP2 protein, while heterozygotes or homozygotes for the risk allele rs10044354 T had increased levels of ERAP2. However, rs10044354 has no relevance to LNPEP and ERAP [60].

#### 4.1.4. Type 1 Diabetes Mellitus (DM1)

Type 1 diabetes mellitus (DM1) is an autoimmune disease characterized by the destruction of insulin-producing pancreatic cells, resulting in elevated blood sugar levels. The development of the disease is influenced by both genetic and environmental factors.

In DM1, there is a frequent association between certain HLA II alleles (HLA-DR3, -DR4, -DQ2, and -DQ8) and the expansion of CD4+ T cells. However, it has been demonstrated that CD8+ T cells also play a significant role in the destruction of pancreatic beta cells. The incorrect processing of epitopes on the surface of the pre-proinsulin protein (PPI) and their subsequent presentation on the HLA-A 02:01 and HLA-A24:02 alleles contribute to the destruction of pancreatic beta cells. This PPI epitope processing mechanism is closely correlated with the activity of ERAP1 [61]. In 2009, Fung et al. demonstrated an association between the ERAP1 rs30187 variant and susceptibility to DM1 [62].

In 2023, Paldino et al. showed the connection between ERAP1, endoplasmic reticulum (ER) stress in pancreatic beta cells, and type 2 diabetes mellitus (DM type 2). The post-transcriptional regulation of ERAP1 in the presence of factors that increase stress in pancreatic beta cells can limit epitope presentation to T lymphocytes. This has been confirmed by Thomaidou et al., who in 2020 showed that the post-translational regulation of ERAP1 leads to the recognition of pre-proinsulin by cytotoxic T lymphocytes. Therefore, both studies suggest that ERAP1 inhibitors could prevent T-cell activation and cytokine production [63,64,65].

#### 4.1.5. Psoriasis (Ps)

Psoriasis is a chronic, immune-mediated inflammatory condition characterized by evolving skin and systemic manifestations. The incidence of psoriasis in the Caucasian population ranges from 2% to 5%. Clinically, it is characterized by the presence of erythematous areas and plaques on various regions of the skin, commonly affecting the scalp, back, elbows, and knees. The disease is primarily driven by the hyperproliferation and hyperkeratinization of keratinocytes. Several types of psoriasis are recognized, including plaque psoriasis, eruptive psoriasis, inverse psoriasis, pustular psoriasis, and erythrodermic psoriasis [66].

Psoriasis is a multifactorial polygenic disease involving both genetic and environmental factors in its pathogenesis manifestations with a prevalence of 0.33–0.6% in different races. About 1,250,000 people are diagnosed with this disease worldwide. Among the genomic regions implicated in disease susceptibility, the PSORS1 gene (psoriasis susceptibility gene 1), located on chromosome 6 (6p21.3), plays a significant role in the genetic determinism of the disease [67,68,69].

There are two types of psoriasis. Type I begins before the age of 40, has a positive family history, and is associated with a high frequency of HLA-C06:02. Type II starts after the age of 40, has a negative family history, and has a normal frequency of HLA-C06:02. While streptococcal angina and stress are triggering factors for Type I, other chronic diseases, and medications trigger Type II. This haplotype mediates the autoimmune response against melanocytes by presenting the autoantigen [70,71]. The ERAP1 gene, through correct trimming of the N-terminus of the peptide required for HLA-C06:02 presentation, contributes to the presentation of the melanocyte autoantigen [72,73].

Key ERAP1 gene polymorphisms involved in mediating the immune response in psoriasis include SNPs: rs26653 (Arg127Pro), rs30187 (Lys528Arg), rs27044 (Gln730Glu), and rs27524. Among these, rs26653 and rs27044 were associated with psoriasis in the Chinese population [74] and rs27044 in Caucasians [75]. The association of rs26653 and rs30187 was found in the early-onset form in the Caucasian population [64,76,77,78].

**Figure 5:**
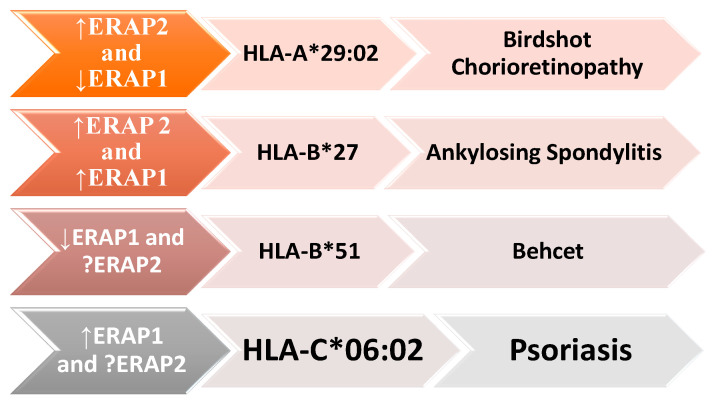
Interrelationship between ERAP1, HLA class I, and autoimmune diseases. * is used in Human Leukocyte Antigen (HLA) nomenclature to denote genetic polymorphisms or allelic variants within the respective HLA gene loci.

It is noteworthy that ERAP1 and ERAP2 are closely related to various HLA class I molecules, including HLA-B27, HLA-C06:02, HLA-A29:02, and HLA-B51, in the pathogenesis of several autoimmune and autoinflammatory diseases. ERAP1′s enzymatic activity can impact the risk of these diseases: when increased, the risk for ankylosing spondylitis and psoriasis is elevated, whereas when it is reduced, the risk decreases for Birdshot chorioretinopathy (BSCR) and Behçet’s disease. The presence of the ERAP2 gene predisposes individuals to ankylosing spondylitis and BSCR (Figure 5) [79].

The interconnectedness between ERAP1, different HLA class I molecules, and the development of autoimmune diseases, including ankylosing spondylitis (AS), psoriasis (Ps), Birdshot chorioretinopathy (BSCR), and Behçet’s disease (BD). These diseases are associated with variants of both ERAP1 and ERAP2, as well as with specific HLA class I molecules, such as HLA-B * 27, HLA-C * 06:02, HLA-A * 29:02, and HLA-B * 51. ERAP1’s enzymatic activity may be increased (up arrow) in some diseases or reduced (down arrow) in others. The role of ERAP2 in psoriasis and Behçet’s disease is still not fully elucidated and requires further research.

### 4.2. Role in Cancer

Analysis of ERAP1 and ERAP2 expression by malignant cells has shown that the levels of these enzymes undergo significant changes that may correlate with the ability of cancer cells to evade immune responses.

The polymorphisms of the ERAP1 and ERAP2 genes play an important role in oncological pathology, and their expression is frequently modified in various forms of cancer, including lung, colon, prostate, kidney, bladder, cutaneous, and hematological cancers. These polymorphisms can cause cancer cells to escape immune responses through different mechanisms:

- Low expression of both enzymes is the most common cause of tumor localization, regardless of its location.
- Downregulation (cutting epitopes too short to be linked to HLA) influences both the nature of the peptides on the cell surface and the cytotoxic response. This occurs in ovarian and breast cancers [80,81].
- Upregulation (tissues with undetectable levels of ERAP1 and ERAP2) is observed in thyroid and colon cancers.
- The balance between ERAP1 and ERAP2 also influences cancer development [10,82].

In cervical cancer, SNPs rs27044, rs26618, and rs26653 in ERAP1 and rs2287988 in ERAP2 influence susceptibility to this tumor form [80]. Mehta et al., in a study of 109 cervical cancer patients, found that ERAP1 expression was elevated in 85% of cases, with 15% showing lower expression levels. Cases with low expression had an unfavorable evolution with rapid metastasis. Additionally, ERAP1 expression is sometimes partially or completely lost in cervical and squamous cell carcinoma [83,84].

Non-small cell lung cancer (NSCLC) constitutes most lung cancer cases, comprising 85–90% of all lung cancer diagnoses [85]. The development of this type of cancer is closely linked to smoking behavior. Wiśniewski et al. conducted a study in the Polish population, revealing significant associations between ERAP1 gene polymorphisms (rs26653, rs2287987, rs30187, and rs27044) and NSCLC, considering both smokers and non-smokers. They found correlations with disease stage (rs27044, PSMB9 rs17587), survival rates (ERAP1 rs30187), and treatment response (ERAP1 rs27044) [86].

Similarly, Yan et al. focused on the Chinese population and underscored the association of specific SNPs of the ERAP1 gene (rs26653G>C, rs26618T>C, rs30187C>T, and rs27044C>G) with the prevalence of NSCLC. These studies highlight the significance of genetic factors in the etiology and clinical outcomes of NSCLC in different populations [87,88].

In melanoma, ERAP1 has a destructive role for many epitopes. Inhibition of ERAP1 increases the binding affinity of HLA class I, decreasing the number of peptides with shorter lengths than normal. Pharmacological inhibition of ERAP1 has been shown to regulate the cellular immunopeptidome [3,89]. Heterogeneous variations of ERAP1 and 2, from high to low levels, were identified in 28 melanoma cell lines compared to primary melanocyte cell lines [90,91].

Medulloblastoma also shows altered expression of ERAP1, indicating its role in the appearance of these tumors [78,92].

Overall, ERAP1 and ERAP2 play crucial roles in cancer, affecting peptide processing and presentation on HLA molecules, thereby influencing the immune response to malignant cells. Understanding these relationships may have implications for potential therapeutic interventions [93].

### 4.3. Atopic Dermatitis

Atopic dermatitis is a chronic inflammatory skin disease with a complex genetic basis. It is characterized by inflammatory skin lesions and persistent itching. The prevalence of atopic dermatitis varies across different regions. Among the genetic factors implicated in the disease, there is an association of HLA C05:01 with ERAP1 polymorphism [94,95,96].

### 4.4. COVID Infection

COVID-19, caused by the SARS-CoV2 virus, belongs to the coronaviridae class (CoVs) and is one of the seven types found in the upper respiratory tract. The infection can range from asymptomatic to severe, leading to multiorgan failure (MSOF). The global impact of COVID-19 has been immense, with millions of reported cases and fatalities [97].

Researchers are extensively studying the viral genome and its correlations with disease severity. The virus binds to angiotensin 2 (ACE2) receptors upon entering the body, which reduces their levels and disrupts the RAS system, resulting in hypertension and increased vascular resistance. ERAP1 and ERAP2 enzymes play crucial roles in the RAS system, and certain polymorphisms may intensify the effects of COVID-19 infection, leading to more severe clinical outcomes and prognosis [98,99].

Recent studies have shown that ERAP1, through its allotypes, contributes to immune responses in COVID-19 infection by modulating the response of CD8+ T cells to viral epitopes [76,100,101]. Moreover, changes in blood pressure during COVID-19 infection are closely correlated with ERAP1 levels. Variants with low enzymatic levels of ERAP1 can elevate angiotensin levels, leading to vascular resistance and hypertension.

### 4.5. Arterial Hypertension (HTA)

Yamamoto et al. conducted a study exploring the association of rs30187 (K528R) in the ERAP gene with essential hypertension. They screened 33 polymorphisms, including 18 new ones, and found that the R528K variant was less active compared to K528R, leading to hypertension due to reduced inactivation of angiotensin II and decreased bradykinin formation from kalidine [101,102]. This variant has also been linked to uremic hemolytic syndrome [103]. Zee et al. demonstrated an association between 33 ERAP1 and 12 ERAP2 polymorphisms with the occurrence of hypertension. They found various genetic variations of ERAP1 associated with incidental hypertension, a progressive increase in blood pressure, and essential hypertension. For example, SNP polymorphisms of ERAP1 rs469783 and rs10050860 were linked to incidental HTA, while SNP ERAP1 rs277772 correlated with a progressive increase in blood pressure [104,105]. Essential hypertension was associated with rs27980 and rs17086651 in a study of patients in northeast China conducted by Yang et al. [17].

## 5. Conclusions and Future Perspectives

*ERAP1* gene polymorphisms have a significant impact on susceptibility to a wide range of conditions related to HLA class I and HLA class II, including autoimmune and autoinflammatory diseases, cancer, infectious diseases, and dermatological disorders. Identifying coding and non-coding variants of ERAP1 allotypes should be a priority to fully understand the role of this gene in human pathology. These insights could contribute to enhancing individualized therapy and guiding medical decision making. Further research in this area holds promise for uncovering valuable insights into disease mechanisms and potential therapeutic targets.

## Figures and Tables

**Table 1:** SNPs linked to AS.

SNPs Polymorphism	Amino Acid Residue Position	Major Allele (Sense Chain)	Minor Allele (Sense Chain)	Mutation (Missense) Allele Major → Allele Minor	Alleles Predisposing to AS
rs30187	528	G	A	AGG → AAG R(Arg) → K(Lys)	Minor allele (coding for Lys, K)
rs27044	730	G	C	GAA → CAA E(Glu) → Q(Gln)	Minor allele (coding for Gln, Q)
rs17482078	725	G	A	CGA → CAA R(Arg) → Q(Gln)	Major allele (coding for Arg, R)
rs10050860	575	G	A	GAC → AAC D(Asp) → N(Asn)	Major allele (coding for Asp, D)
rs2287987	349	A	G	ATG → GTG M(Met) → V(Val)	Major allele (coding for Met, M)
